# On the Subcutaneous Use of Ergotine in Diabetes and Albuminuria

**Published:** 1888-01

**Authors:** 


On the Subcutaneous Use of Ergotine in Diabetes and Albuminuria.—A. Dehenne (L'Union Med.) claims to demonstrate:

1. That ergotine, or ergotinine, subcutaneously will cause the temporary and often the permanent disappearance of the glycosuria, polydipsia, polyuria, emaciation, and weakness of diabetes.

2. That these symptoms disappear in a regular order; the polyuria and polydipsia disappear after five to eight injections; the glycosuria lessens after the second or third injection, and disappears after the tenth or twelfth.

3. That the glycosuria reappears if the treatment be stopped too suddenly.

4. That the disappearance is permanent after six or eight weeks of treatment.

5. That the injections are perfectly harmless.

6. That by this treatment diabetics can be prepared for any surgical operation, particularly cataract.

7. The freedom of this treatment from digestive disturbances.

He injects six to ten drops, sometimes more, daily.—*Kansas City Medical Index.*

---

*The Popular Science News*, on the subject of impure ice, which has lately been much discussed in medical journals, says: Freezing is a process of crystallization, and the tendency is always to eliminate impurities from the crystals; but the foreign matters can and do remain entangled in the mass among the intercrystalline spaces. There have been undoubted cases of typhoid fever and other diseases produced from this cause, and it is a safe rule only to use ice formed from water of assured purity. In other words, wholesome ice cannot be made from unwholesome water.

